# Two new species of the rare Neotropical caddisfly genus *Amphoropsyche* Holzenthal (Trichoptera, Leptoceridae)

**DOI:** 10.3897/zookeys.707.20759

**Published:** 2017-10-10

**Authors:** Ernesto Rázuri-Gonzales, Ralph W. Holzenthal, Blanca Ríos-Touma

**Affiliations:** 1 University of Minnesota, Department of Entomology, 1980 Folwell Ave., 219 Hodson Hall, St. Paul, Minnesota 55108 U.S.A.; 2 Universidad de Las Américas, Facultad de Ingenierías y Ciencias Agropecuarias, Ingeniería Ambiental, Campus Queri, Calle José Queri, Edificio #8, PB, Quito, Ecuador

**Keywords:** Endemic, new species, taxonomy, Ecuador, Peru, Neotropics, male genitalia, Andes, South America

## Abstract

Two new species in the rare, endemic Neotropical caddisfly genus *Amphoropsyche* Holzenthal, 1985 are described from Ecuador (*A. carchi* **sp. n.**) and Peru (*A. matsigenka* **sp. n.**) bringing to 17 the number of species known in the genus. Almost all species are known from only a few individuals and from even fewer localities. The new species belong to a group of 10 other species that have tergum X in the male genitalia divided into a mesal process and a pair of lateral processes. *Amphoropsyche carchi* can be separated from those species by the rounded mesal concavity, the short mesobasal lobe, and the short 2nd article of the inferior appendage, while *A. matsigenka* can be diagnosed by the very slender and straight inferior appendage, which bears a pair of spine-like mesoventral projections. We also present a new record for *Amphoropsyche tandayapa* Holzenthal & Rázuri-Gonzales, 2011, from Ecuador, previously known only from the male holotype.

## Introduction

The recently published *Catalog of the Neotropical Trichoptera* lists more than 3,200 species occurring in the region of Mexico, the Caribbean, and Central and South America (Holzenthal and Calor 2017). This figure represents an increase in more than 1,000 new species described since the last catalog of the fauna was published, about 20 years ago (Flint et al. 1999). New species continue to be described, especially from unexplored areas (e.g., Quinteiro and Holzenthal 2017; Souza and Santos 2017) indicating that the caddisfly fauna of the Neotropics holds many more species.

In this paper, we add two new species in the genus *Amphoropsyche* Holzenthal, 1985, a member of the long-horned caddisfly family Leptoceridae, to the Neotropical fauna. The family contains more than 221 species in the Neotropics. Eight of the 16 genera occurring in the region are endemic (Holzenthal and Calor 2017). The endemic genus *Amphoropsyche* currently includes 15 species (one further divided into two subspecies), 13 from South America, and the remaining from the Lesser Antilles (Holzenthal and Calor 2017). All these species are very rare; individuals are only very infrequently attracted to UV lights or caught in Malaise traps, standard methods for collecting adult caddisflies, and most of the described species are known from only one or two specimens (Holzenthal and Rázuri-Gonzales 2011, Holzenthal and Ríos-Touma 2016). On the other hand, Flint and Holzenthal observed a large swarm of individuals of *Amphoropsyche woodruffi* Flint & Sykora, 1993 flying during the day above a small shallow stream in Venezuela, indicating that the species in the genus may be day active and not as rare as suggested from light trap collections alone. The larva of only one species is known (Holzenthal 1986) and its life history is unknown. Knowledge of adult behavior is non-existent, except for the swarming behavior observed for *A. woodruffi*. The two new species described in this paper from Ecuador and Peru are known from only 3 individuals, and no biological information was observed.

## Material and methods

The Ecuadorian specimen was collected during an ongoing project by the authors and their colleagues to document the Trichoptera fauna of the country while the Peruvian specimens were collected during an inventory carried out prior to the exploitation of the Camisea natural gas reserve in southeastern Peru. Specimens were either collected using UV lights (Ecuadorian specimen) or a Malaise trap (Peruvian). Techniques and procedures used in the preparation and examination of the specimens were outlined by Blahnik and Holzenthal (2004) and Blahnik et al. (2007). The illustrations of the genitalia were prepared from pencil sketches made with the aid of a drawing tube mounted on an Olympus BX41 compound microscope. The pencil sketches were then scanned and placed into an Adobe Illustrator (version CC, Adobe Systems, Inc.) document, to serve as a template, and then digitally drawn to create a vector graphic illustration. A graphic tablet and pen (Intuous TM, Wacom Technology Co.) facilitated careful drawing of the original image.

Terminology used in describing male and female genitalia follows that of Holzenthal and Ríos-Touma (2016). The types will be deposited in the Museo Ecuatoriano de Ciencias Naturales, Quito, Ecuador (MECN) and the Museo de Historia Natural, Universidad Nacional Mayor de San Marcos, Lima, Peru (MUSM), as specified below. Each specimen bears an accession number from the University of Minnesota Insect Collection and the specimen and collection data are stored in the collection’s database.

## Systematics

### Amphoropsyche carchi

Taxon classificationAnimaliaTrichopteraLeptoceridae

Rázuri-Gonzales, Holzenthal & Ríos-Touma sp. n.

http://zoobank.org/B82E8DB8-2F36-4F10-9898-74BED8262B35

Fig. 1

#### Diagnosis.

This species is diagnosed by the structure of the inferior appendage and the phallic apparatus, especially the rounded mesal concavity, the short mesobasal lobe and the short 2nd article of the inferior appendage, and the phallobase with paired apicolateral projections each bearing a short, apical spine-like seta. This new species belongs to a group of 10 species whose males have segment X divided into a mesal process and a pair of lateral processes (*A. ayura* Holzenthal, 1985, *A. cauca* Holzenthal, 1985, *A. choco* Holzenthal, 1985, *A. flinti* Holzenthal, 1985, *A. napo* Holzenthal, 1985, *A. quebrada* Holzenthal, 1985, *A. real* Holzenthal & Ríos-Touma, 2016, *A. spinifera* Holzenthal, 1986, *A. stellata* Holzenthal, 1985, and *A. tandayapa* Holzenthal & Rázuri-Gonzales, 2011). Among these, *A. carchi* sp. n., is most similar to *A. quebrada* based on the structure of the inferior appendage (short 2nd segment, deep mesal concavity in ventral view), but the mesal notch on the preanal appendage of the new species is not as deep as in *A. quebrada*. Additionally, *A. carchi* lacks parameres in the phallic apparatus, but has a pair of apicolateral projections each with an apical spine-like seta, and the lateral process of tergum X in the new species lacks any spine-like setae apically. In the key to males of the genus provided by Holzenthal and Ríos-Touma (2016), the new species will lead to *A. spinifera*, but the paired apicolateral processes of the phallobase are not bifid as they are in *A. spinifera*.

#### Description.

**Figure 1.**
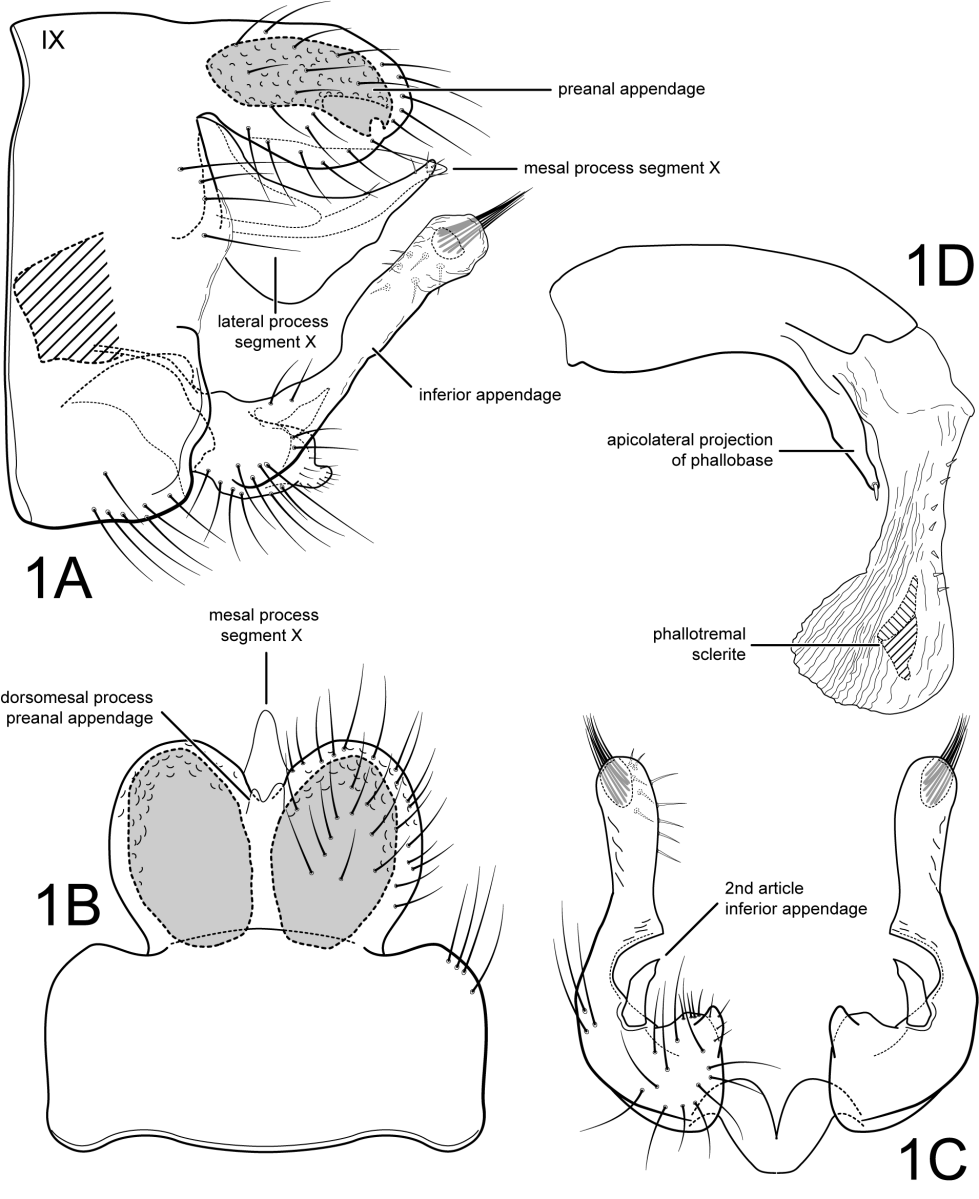
*Amphoropsyche carchi*, new species. Male genitalia **A** segments IX-X, lateral **B** segments IX-X, dorsal **C** inferior appendages, ventral **D** phallus, lateral. IX=abdominal segment IX.

Male. Forewing length 5.5 mm (n=1). Body and legs brown, no discernable color pattern, antennae cream colored (specimen pinned, base of wings slightly denuded). Genitalia as in Fig. 1A–D. Segment IX annular, sternum with anterior margin straight not extended anteriorly (Fig. 1A). Segment X composed of single mesal process and pair of lateral processes (Fig. 1A–B); mesal process very lightly sclerotized, slightly longer than preanal appendages, triangular, apex acute (Fig. 1B); lateral process broadly crescent-shaped, without any spine-like setae; apically, lateral process with about 5-6 small setae (Fig. 1A). Preanal appendages large, oval, fused basally but divided apically to 1/3 their lengths (apical emargination broad); with large reticulate internal gland and small subapicoventral pore (Fig. 1A–B); apically with pair of short, digitate, membranous dorsomesal processes (Fig. 1B). Inferior appendage narrow, elongate, bent dorsad from base, with short basoventral projection bearing short setae (Fig. 1A, C); in ventral view, with deep, rounded mesal concavity with sharp posterior angle; ending in bulbous, semi-membranous apex, bearing subterminal tuft of closely appressed setae emerging from membranous pocket and with prominent setae on mesal face; 2nd article of inferior appendage short, thin, slightly curved inwards in ventral view, apex acute; 2nd article sitting on ridge at base of mesal concavity (Fig. 1C). Phallic apparatus (Fig. 1D) with phallobase well-developed, with sclerotized apicolateral projection on each side, bearing stout apical spine-like seta; parameres absent; endothecal membranes well-developed, everted on specimen, with row of small spicules anterodorsally; phallotremal sclerite U-shaped, well-developed, triangular in lateral view.

#### Holotype.

**Male. ECUADOR: Carchi**: Quebrada San Francisco (Hacienda San Francisco), ca. 1.8 km W Las Juntas, 00.80330°N, 78.17081°W, el. 1241 m, 15.ii.2017, Ríos-Touma & Amigo (UMSP000114269) (MECN).

#### Etymology.

This species is named after the Province of Carchi, where the type was collected.

### Amphoropsyche matsigenka

Taxon classificationAnimaliaTrichopteraLeptoceridae

Rázuri-Gonzales, Holzenthal & Ríos-Touma sp. n.

http://zoobank.org/6444085A-E7FC-455D-A4B5-FC42254375B5

Figs 2 , 3

#### Diagnosis.

This species is mainly diagnosed by the structure of the inferior appendage, the phallic apparatus, and the mesal process of tergum X. The inferior appendage is very slender and straight and bears a pair of spine-like mesoventral projections. One or both of these spine-like mesoventral projections could represent the 2nd article that has become fused to the body (1st article) of the inferior appendage; however, while these are positioned where the 2nd article occurs in those species that possess one, no indication of articulation or fusion is apparent). The phallic apparatus has a strongly sclerotized apicolateral projection of the phallobase and the endothecal membranes have a pair of lightly sclerotized spine-like projections dorsally. Finally, the mesal process of segment X is elongate, talon-like, curved ventrad apically, and much longer than the preanal appendages. As with *A. carchi* species, this new species is related to the group of species with divided tergum X. It resembles *A. spinifera*, due to the very slender and almost straight inferior appendage, but differs in the spine-like mesoventral projections (if these are interpreted to be the 2nd article, their structure is different). The lateral processes of tergum X are also similar between the 2 species, but the mesal processes are quite different; in *A. spinifera* the process is only slightly shorter than the preanal appendages and the apex is rounded, but in *A. matsigenka* it is much longer than the preanal appendages and the apex is sharply pointed. In the key to males of the genus provided by Holzenthal and Ríos-Touma (2016), *A. matsigenka* will lead to couplet 14 containing *A. cauca* and *A. ayura* if the 2nd article of the inferior appendage is assumed absent, but the new species lacks the phallic parameres found in those species.

#### Description.

**Figure 2.**
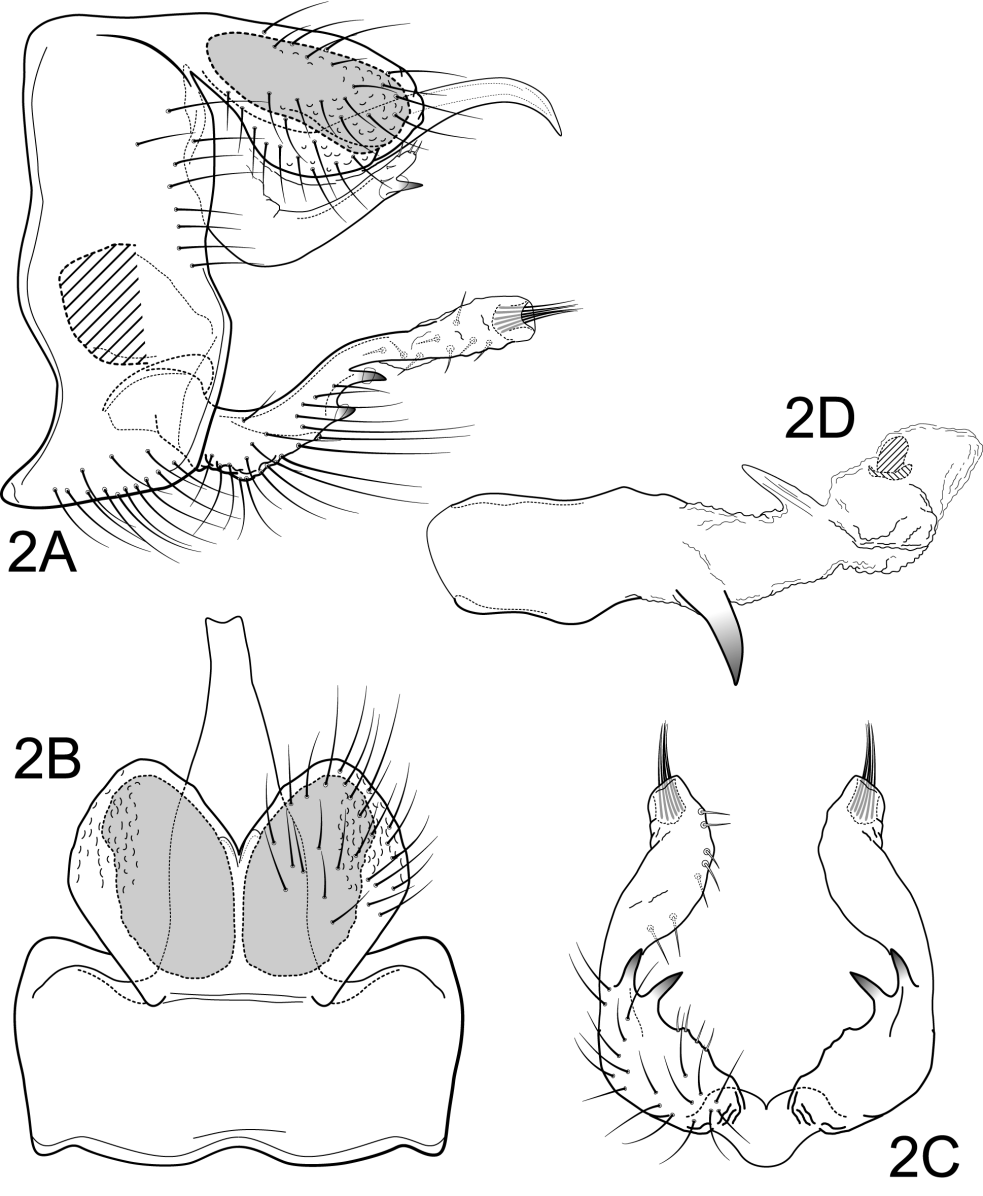
*Amphoropsyche matsigenka*, new species. Male genitalia **A** segments IX-X, lateral **B** segments IX-X, dorsal **C** inferior appendages, ventral **D** phallus, lateral.

Male. Forewing length 5.5 mm (n=1). Body and appendages brown (specimen preserved in 80% ethyl alcohol, wings denuded). Genitalia as in Fig. 2A–D. Segment IX annular, sternum with anterior margin slightly extended anteriorly (Fig. 2A). Segment X composed of single mesal process and pair of lateral processes (Fig. 2A–B); mesal process elongate, talon-like, curved ventrad apically, and much longer than preanal appendage; lateral process broadly crescent-shaped, with spine-like seta subapically on ventral margin; apically lateral process with ca. 5 small setae (Fig. 2A). Preanal appendages large, oval, fused basally but divided apically to 1/2 their lengths (apical emargination acute); with large reticulate internal gland and small subapicoventral pore (Fig. 2A–B); apically with pair of very reduced, digitate, membranous dorsomesal processes (Fig. 2B). Inferior appendage narrow, elongate, almost straight, with pair of spine-like mesoventral projections (Fig. 2A, C); in ventral view, concave mesally; ending in bulbous, semi-membranous apex, bearing subterminal tuft of closely appressed setae emerging from membranous pocket and with prominent setae on mesal face; 2nd article of inferior appendage absent [or one or both of the spine-like mesoventral projections could represent the 2nd article that has become fused to the first article] (Fig. 2C). Phallic apparatus (Fig. 2D) with phallobase well-developed, with sclerotized apicolateral projection on each side, without apical spine-like seta; parameres absent; endothecal membranes well-developed, everted on specimen, with pair of lightly sclerotized spine-like projections dorsally; phallotremal sclerite U-shaped, well-developed, oval in lateral view.

**Figure 3.**
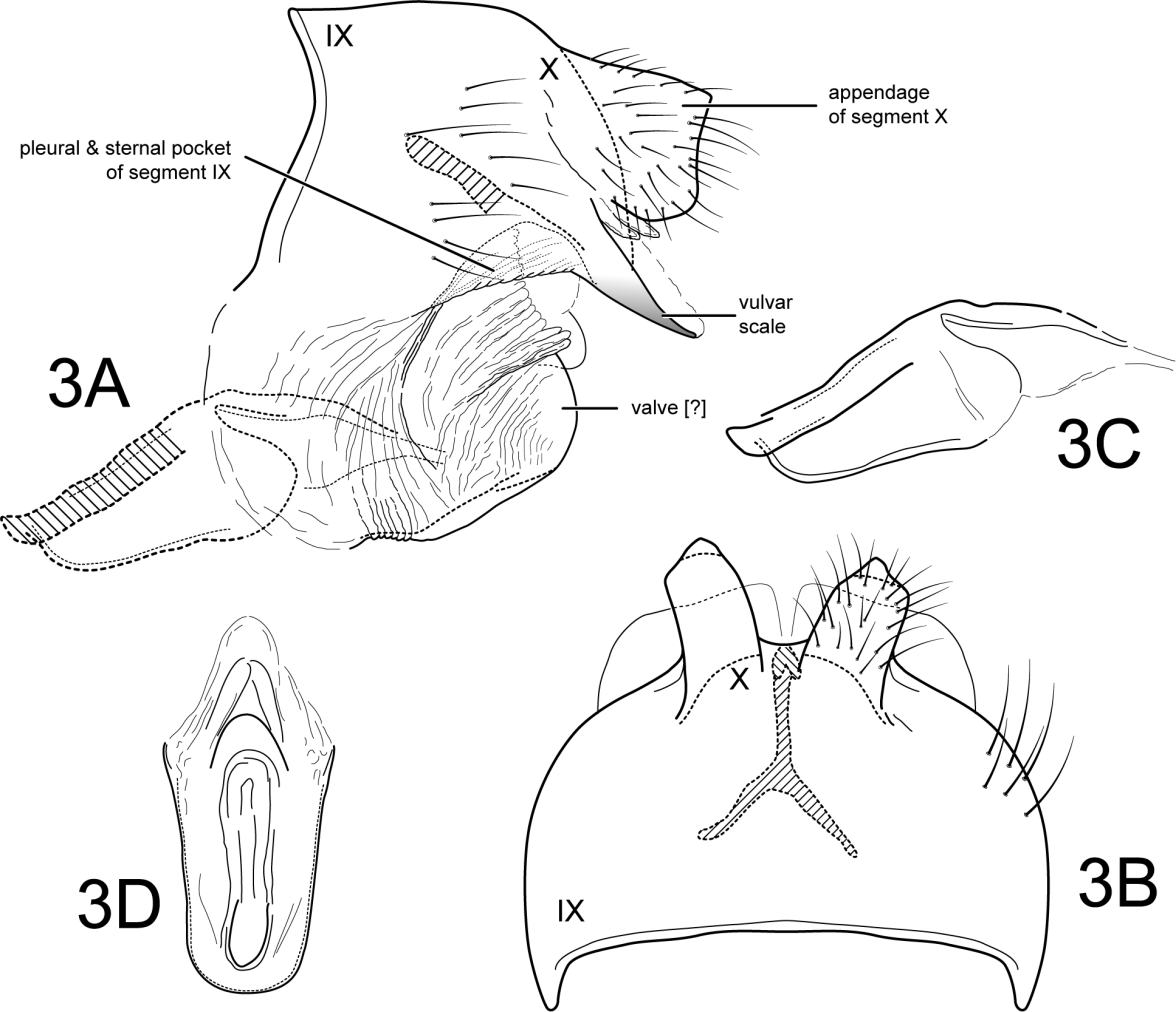
*Amphoropsyche matsigenka*, new species. Female genitalia **A** segments IX-X, lateral **B** segments IX-X, dorsal **C** vaginal apparatus, lateral **D** vaginal apparatus, ventral. IX=abdominal segment IX, X=abdominal segment X.

Female. Forewing length 5.5 mm (n=1). Color and structure similar to male’s (specimen preserved in 80% ethyl alcohol, wings denuded). Genitalia as in Fig. 3A–D. Abdominal tergum IX + X complete; dorsomesal margin entire; distinctive Y-shaped sclerite internally. Appendages of segment X quadrate, longer than wider, setose, directed laterad. Valves posterolateral, rounded, wider than longer, bare, but with highly folded membranous surface and prominemt dorsal ridge of folded membrane [these structures are less evident than the valves in other species so their homology as valves is uncertain]. Vulvar scale thin, more sclerotized than surrounding tissues, narrow in lateral view, round in dorsal view with slight mesal excavation. Pleuron region and sternum IX laterally forming large, prominent pocket-like structure. Vaginal apparatus (spermathecal sclerite complex) (see Fig. 3C, D) with broad, oval, posterior base bearing central “keyhole-shaped” structure; middle region to apex with narrow lightly sclerotized plates and membrane.

#### Holotype.

**Male. PERU: Cusco**: La Convención, Echarate, Cashiriari-3 [Shell prospecting and development project], 11.86667°S, 72.65°W, el. 690 m, xi-xii.1997, S. Córdoba, (UMSP000114270) (MUSM). **Paratypes**: Same data as holotype, 1 female (UMSP000114271) (MUSM).

#### Etymology.

This species is named after the Matsigenka ethnic group, whose communities are spread throughout the departments of Cusco and Madre de Dios in southeastern Peru, more specifically, the Cashiriari community that inhabits the area where the holotype was collected.

### Amphoropsyche tandayapa

Taxon classificationAnimaliaTrichopteraLeptoceridae

Holzenthal & Rázuri-Gonzales, 2011

urn:lsid:zoobank.org:act:405CE4BA-B14D-4CEB-827E-BFE295FD12F0

#### New record.

**ECUADOR: Pichincha**: Bellavista Cloud Forest Reserve and Lodge, small stream, pan trap, 0.01212°S, 78.68958°W, 13–14.vii.2017, el. 2614 m, col. Andrea Tapia, 2 males (MECN).

## Discussion

*Amphoropsyche matsigenka* was collected in the Urubamba River basin, which eventually drains into the Amazon River in northeastern Peru. This new species was collected some 170 km northwest of the only other species known from the country (*A. spinifera*; Flint 1996), which appears to be restricted to the Beni River basin, which also flows into the Amazon in northern Brazil. Conversely, *A. carchi* is only the 2nd species of *Amphoropsyche* known from the western slope of the Andes (*A. tandayapa* is the other), and these 2 species are separated from each other by roughly 100 km. However, the rivers on the Pacific side of the Andes are shorter and do not form extensive watersheds like those on the Amazon slopes, but they can be much more complex. These smaller western watersheds are isolated from one another by the topographical complexity of the Andes and this could have acted as barriers that allowed these 2 species to diverge. On the other hand, the record of *A. spinifera* from the Amazonian slope of Bolivia (Holzenthal 1986) is roughly 956 km SE of the record of the same species from Manu, Peru, indicating that the Amazonian species in the genus are not as isolated and may be more widespread, but probably still restricted to particular basins.

The rarity of the members of this genus limits the study of their diversity, habitat preferences, and life history features, and to date, all additional individuals collected in the northern Andes, on either slope, since Holzenthal’s (1985) revision have been new species. *Amphoropsyche matsigenka* is only the 2nd species collected from the large country of Peru since Flint’s (1996) record of *A. spinifera*. Similarly, *A. carchi* is the 4th species recorded or described from Ecuador. Even with our recent concentrated efforts to expand the knowledge of Ecuadorian Trichoptera (Ríos-Touma et al. 2017) and our studies in Peru, the localities and the number of individuals known for *Amphoropsyche* are very limited. Given the apparent localized distribution of the species in this genus, it is highly probable that any specimens of this genus discovered in the future will be new to science, especially with increased sampling effort and the exploration of new regions in the other northern and central Andean countries (Bolivia, Colombia, and Venezuela).

## Supplementary Material

XML Treatment for Amphoropsyche carchi

XML Treatment for Amphoropsyche matsigenka

XML Treatment for Amphoropsyche tandayapa
